# Permeability‐Engineered Compartmentalization Enables In Vitro Reconstitution of Sustained Synthetic Biology Systems

**DOI:** 10.1002/advs.202203652

**Published:** 2022-09-30

**Authors:** Luyao Li, Rong Zhang, Long Chen, Xintong Tian, Ting Li, Bingchun Pu, Conghui Ma, Xiangyang Ji, Fang Ba, Chenwei Xiong, Yunfeng Shi, Xianqiang Mi, Jian Li, Jay D. Keasling, Jingwei Zhang, Yifan Liu

**Affiliations:** ^1^ School of Physical Science and Technology ShanghaiTech University Shanghai 201210 China; ^2^ State Key Laboratory of Genetic Engineering School of Life Sciences Fudan University Shanghai 200438 China; ^3^ Department of Immunology and Microbiology Shanghai Jiao Tong University School of Medicine Shanghai 200025 China; ^4^ Shanghai Institute of Microsystem and Information Technology Chinese Academy of Sciences Shanghai 200050 China; ^5^ Joint BioEnergy Institute Emeryville CA 94608 USA; ^6^ Biological Systems and Engineering Division Lawrence Berkeley National Laboratory Berkeley CA 94720 USA; ^7^ Department of Chemical and Biomolecular Engineering & Department of Bioengineering University of California Berkeley CA 94720 USA; ^8^ Shanghai Clinical Research and Trial Center Shanghai 201210 China

**Keywords:** biomaterials, cell‐free protein synthesis, compartmentalization, synthetic biology, whole‐cell biosensors

## Abstract

In nature, biological compartments such as cells rely on dynamically controlled permeability for matter exchange and complex cellular activities. Likewise, the ability to engineer compartment permeability is crucial for in vitro systems to gain sustainability, robustness, and complexity. However, rendering in vitro compartments such a capability is challenging. Here, a facile strategy is presented to build permeability‐configurable compartments, and marked advantages of such compartmentalization are shown in reconstituting sustained synthetic biology systems in vitro. Through microfluidics, the strategy produces micrometer‐sized layered microgels whose shell layer serves as a sieving structure for biomolecules and particles. In this configuration, the transport of DNAs, proteins, and bacteriophages across the compartments can be controlled an guided by a physical model. Through permeability engineering, a compartmentalized cell‐free protein synthesis system sustains multicycle protein production; ≈100 000 compartments are repeatedly used in a five‐cycle synthesis, featuring a yield of 2.2 mg mL^−1^. Further, the engineered bacteria‐enclosing compartments possess near‐perfect phage resistance and enhanced environmental fitness. In a complex river silt environment, compartmentalized whole‐cell biosensors show maintained activity throughout the 32 h pollutant monitoring. It is anticipated that permeability‐engineered compartmentalization should pave the way for practical synthetic biology applications such as green bioproduction, environmental sensing, and bacteria‐based therapeutics.

## Graphical abstract

A permeability‐engineerable compartmentalization strategy is presented to enable the design and construction of sustained and robust synthetic biology systems. Hydrogel‐based core–shell compartments are fabricated via high‐throughput microfluidics and show controllability over the transport of biomolecules and particles through permeability adjustment. The strategy leads to recyclable cell‐free protein synthesis and compartmentalized whole‐cell biosensors that feature enhanced environmental fitness.

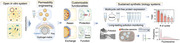

## 1 Introduction

Compartmentalization is a widespread phenomenon among biological systems.^[^ ^1^, ^2^, ^3^, ^4^ ^]^ Cells are compartmentalized, preventing the loss of biochemical intermediates and possessing reduced entropy and increased robustness. Meanwhile, cells must be permeable in order to gain sustained exchange of matters.^[^ ^5^ ^]^ Thus, they exhibit an manipulated permeability to the environment through various passive and active routes.^[^ ^6^ ^]^ Relegating the degree of permeability is also crucial for cell adaptability. The permeability of bacteria cell membranes is dynamically tuned to resist environmental impacts.^[^ ^7^, ^8^, ^9^ ^]^ The maintained and precisely controlled permeability of biological compartments contributes to the sustainability and complexity of biological systems.

Over the years, efforts have been devoted to reconstituting cell‐like compartments in vitro, aiming to build cell‐mimic systems^[^ ^10^, ^11^, ^12^ ^]^ and perform digitalized reactions.^[^ ^13^, ^14^ ^]^ A typical strategy is water‐in‐oil emulsification, which yields isolated aqueous compartments surrounded by an immiscible oil phase,^[^ ^15^ ^]^ yet the surrounding oil hinders matter exchange in such a two‐phase system. In parallel, researchers have created synthetic‐membrane‐based compartments, which are selectively permeable.^[^ ^16^, ^17^, ^18^ ^]^ However, these compartments are inherently fragile, limiting their robustness in practical applications. Different strategies (e.g., use DNA or protein pores) have been applied to manipulate their permeability but required laborious efforts.^[^ ^19^, ^20^, ^21^ ^]^ Hydrogel is a kind of biocompatible and intrinsically porous materials, which have been widely used to accommodate biomolecules and cells.^[^ ^22^, ^23^ ^]^ Hydrogel‐based compartments such as gel beads and capsules provide a semipermeable aqueous environment, enabling matter exchange desired for biochemical reactions and cell growth. Nevertheless, such systems have not yet explored the control of permeability and its usefulness in engineering in vitro systems. A simple and robust strategy of permeability‐controlled compartmentalization is still to be developed, which could significantly expand the robustness, function, and complexity of compartmentalized in vitro systems.

**Figure 1:**
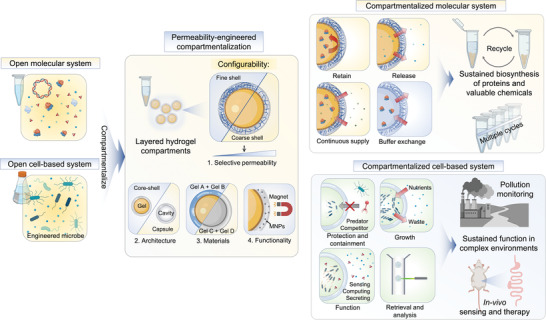
Schematic of the permeability‐engineered compartmentalization strategy. PeCS compartmentalizes open in vitro molecular and cell‐based systems in micrometer‐sized, layered hydrogel‐based compartments. The compartments own configurability in selective permeability, architecture, materials, and functionalization. The compartmentalized systems allow biological reactions and activities to execute in a sustained manner due to engineered passive transport. For a reconstituted molecular system, the selective permeability of compartments can be tuned to achieve desired transport of biomolecules such as protein and DNA while allowing constant input of reactants and energy, enabling sustained biosynthesis. Reconstituted cell‐based systems can protect the encapsulated cells from competitors and predators and support the cells’ growth and functioning, thereby enhancing the fitness of these functional synthetic biology systems in complex environments such as polluted water and animal guts.

Here we describe a permeability‐engineered compartmentalization strategy (PeCS) and demonstrate its utility in reconstituting synthetic biology systems with significantly enhanced robustness and sustainability. PeCS inherits the advantages of hydrogel encapsulation and, beyond that, allows for precise control of permeability and thus passive transport. The strategy uses microfluidics to produce micrometer‐sized, layered hydrogel compartments (**Figure** **1**). The outer layer serves not only as a simple physical barrier but a sieving structure that is semipermeable to biomolecules and particles. Notably, its permeability can be tuned by adjusting the pore size, allowing for customizable retaining/releasing. The impact of adjusted pore size on the transport behavior of biomolecules such as protein and DNA can be guided by a modified Kratky–Porod model.^[^ ^24^ ^]^ Other than permeability, PeCS is flexible in compartment architecture (hollow capsule or core–shell bead), material composition, and functionality. For instance, the core material is switchable: when encapsulating cells, a cell‐friendly hydrogel can be chosen to facilitate cell growth and function; to encapsulate molecular assays, the core can be selectively dissolved, leaving a liquid cavity ideal for biochemistry.

In this work, we explore the utility of PeCS in synthetic biology and demonstrate permeability‐engineered compartmentalization of 1) a cell‐free protein synthesis (CFPS) system and 2) bacteria whole‐cell biosensors to address their current limitations. CFPS enables easy, rapid, and controllable protein expression by breaking the physical boundary of cells.^[^ ^25^, ^26^ ^]^ Meanwhile, however, the lack of compartmentalization in the homogeneous system suggests that it is not sustained as the DNAs and enzymes cannot be retained and reused.^[^ ^27^ ^]^ Through permeability engineering, the PeCS‐based CFPS system bridges in vivo transcription and translation (TX–TL) and open‐environment CFPS; it retains the gene transcription in the compartments whereas the translation events occur in the outer solution. After the completion of protein synthesis, the compartments (and the encapsulated genes and enzymes) can be magnetically purified and added to a fresh feeding solution to initiate a new round of CFPS. We demonstrate that PeCS‐based CFPS supports a five‐cycle synthesis of superfolder green fluorescent protein (sfGFP) with a total yield of over 2.2 mg mL^−1^. On the other hand, we show that permeability engineering can be applied to living bacteria whole‐cell biosensors to improve their environmental fitness. Compartmentalized whole‐cell biosensors prepared by PeCS show near‐perfect resistance against wild bacteriophages and sustained functionality. We further demonstrate that PeCS enables the deployment and retrieval of biosensor compartments to monitor pollutant levels in river silt samples and high‐throughput analysis to retrieve recorded pollutant information by flow cytometry.

## 2 Results

### 2.1 Manufacturing of PeCS Compartments

**Figure 2:**
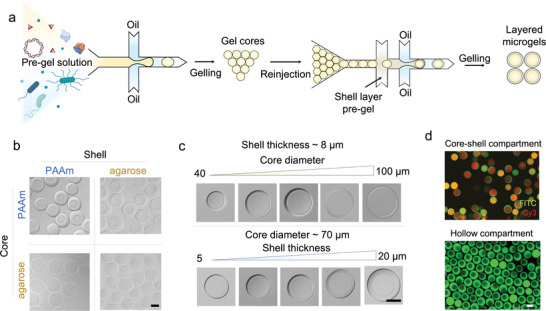
Manufacturing of the PeCS compartments. a) Microfluidic workflow to encapsulate synthetic biology systems in layered microgels. The systems are pre‐encapsulated in the hydrogel cores using a microfluidic dropmaker. The core beads are further injected to a second microfluidic device to generate layered microgels. b–d) Optical microscopic images of b) compartments composed of arbitrary combinations of polyacrylamide (PAAm) and agarose hydrogels, c) compartments featuring tunable core diameters from 40 to 100 µm and shell thickness from 5 to 20 µm, and d) core–shell hydrogel compartments and hollow compartments after core dissolving. The core and shell layers of compartments in panel (d) are fluorescently labeled with Cy3 (red) and fluorescein isothiocyanate‐dextran (green), respectively. Scale bars: 50 µm.

The PeCS strategy compartmentalizes in vitro systems within layered microgels through a two‐step droplet microfluidic approach (**Figure** **2**a). First, molecular assays or cell solutions containing hydrogel precursors are emulsified to picoliter droplets in a flow‐focusing microdevice (Figure S1a, Supporting Information). The generated droplets are gelled and de‐emulsified to obtain monodispersed gel cores. Then, the core beads are reinjected into a dual‐junction flow‐focusing device (Figure S1b, Supporting Information) to build the shell layer. Due to the close‐packed nature^[^ ^28^ ^]^ of gel beads in microchannels, core–shell hydrogel droplets are generated at ≈90% efficiency (Figure S2, Supporting Information). Gelling and demulsification are repeated to obtain the final layered hydrogel compartments. The procedure can be completed in 3 h, yielding over 3000 000 compartments.

### 2.2 Configuring Composition and Architecture of Compartments

The two‐step workflow of PeCS offers the ease of altering core and shell hydrogel materials on demand. Figure 2b shows manufactured core–shell compartments composed of arbitrary combinations of polyacrylamide (PAAm) and agarose hydrogels. PAAm gels are typically dense in polymer networks, thus favorable for sieving biomolecules. Agarose gel features mild radical‐free polymerization, thus being more friendly to accommodate living cells. Moreover, the workflow allows precise tuning of the compartment geometry (Figure 2c). The core diameter and shell thickness are adjustable between 40–100 and 5–20 µm, respectively, by simply modifying the microfluidics. If desired, the cores can be selectively dissolved to create a liquid cavity ideal for biochemical reactions. Figure 2d displays the manufactured compartments featuring a core–shell architecture with each layer being labeled by distinct fluorescence. The core gel is reversibly crosslinked using a disulfide bond linker, *N*,*N*′‐*bis*(acryloyl)cystamine, which can be chemically dissolved with the assistance of a reducing agent such as dithiothreitol (Figures S3 and S4, Supporting Information), leading to a hollow architecture. Moreover, the compartments can be conveniently magnetized by encapsulating ferric oxide nanoparticles (NPs) in the shell, enabling efficient bead purification and buffer exchange (Figure S5, Supporting Information).

### 2.3 Engineering Selective Permeability of Compartments

**Figure 3:**
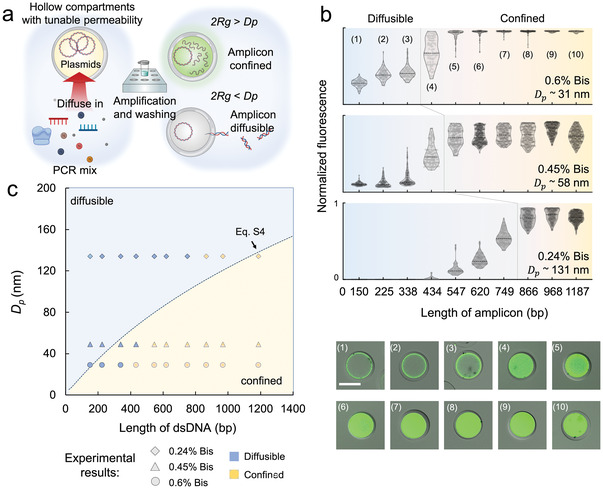
Engineering selective permeability of the PeCS compartments. a) Schematic of a compartmentalized PCR assay designed to explore the selective permeability of compartments. If the dimension of amplicon DNA (double of its gyration radius *R*_g_) is greater than the pore size of compartment shell (*D*_p_), they are expected to be accumulated in the cavity. By contrast, smaller amplicons will diffuse to the outer solution. Consequently, smaller amplicons can be removed from the compartment cavities and the outer solution in the washing steps, where larger amplicons are still trapped in the cavities. The process can be visualized by fluorescent labeling of the amplicons. b) Results of compartmentalized PCR performed in compartments with configured pore sizes. The scatter plots (top) display the fluorescence profile (normalized) of the compartments subjected to serial PCRs. Each point in the plots represents the value of a single compartment (*n* ≥ 50 for each set of data). The series of fluorescent microscopic images (bottom) show individual 0.6% Bis–PAAm compartments undergone corresponding PCR conditions. Scale bars: 50 µm. c) Physical model describing the transport characteristic of double‐stranded DNA (dsDNA) across the compartments. The scattered points represent experimental results (legend) obtained from the compartmentalized PCR.

The selective permeability of hydrogels has extensively been utilized to sieve biomolecules.^[^ ^29^ ^]^ We hypothesized that the hydrogel shell of PeCS compartments could serve as a similar molecular sieve, selectively barring the transport of biomolecules on our demand. To test our hypothesis, we designed a PeCS‐based polymerase chain reaction (PCR). As illustrated in **Figure** **3**a, DNA plasmids are pre‐encapsulated in the cavities (≈110 pL in volume) of hollow compartments (≈75 µm in diameter) as PCR templates. They are assumed to be physically confined therein. A PCR mix is externally introduced and infused into the compartments. Suppose that the physical dimension of amplicon DNA (double of its gyration radius *R*_g_) is greater than the critical pore size of the hydrogel shell (*D*_p_). In this case, they are expected to accumulate in the cavity during the amplification, whereas smaller products are rather free to transport to the outer environment. Therefore, in subsequent washing procedures, smaller amplicons should be removed from the compartment cavities and the outer solution, where larger amplicons would still be trapped in the cavities. A preliminary experiment was first conducted, confirming that our customized PCR strategy was conducted as designed (Figure S6, Supporting Information). We then manufactured a set of compartments with three distinct shell pore sizes. This was accomplished primarily by tuning the ratio of crosslinker, bis‐acrylamide (Bis), of a PAAm gel (Tables S2 and S3, Supporting Information). Determined by scanning electron microscopy, the average *D*_p_values of PAAm gels containing 0.6%, 0.45%, and 0.24% Bis are 29, 49, and 134 nm, respectively (Figure S7, Supporting Information). The pore size profile aligns with previous reports characterizing the PAAm gels.^[^ ^30^, ^31^ ^]^

We next designed ten primer pairs targeting amplicons ranging from 150 to 1187 bp on the plasmid and performed a series of PeCS‐based PCRs (Figure S8, Supporting Information). Figure 3b displays the fluorescence distribution of compartments (Figure S9, Supporting Information) subject to various PCR conditions and a set of microscopic images of 0.6% Bis–PAAm compartments post PCR. Overall, with the increase in their length, the DNA amplicons undergo a transition from 1) being diffusible through the shell (low fluorescence) to 2) being confined in the cavity (high fluorescence). For each different *D*_p_, however, the transition occurs at distinct stages. The threshold lengths at which most of the amplicons are trapped are estimated to be ≈ 434, 547, and 866 bp for *D*_p_ values of 31, 58, and 131 nm, respectively, showing a positive correlation with *D*_p_. These results verify that the compartments indeed possess a size selection effect of DNA molecules, and, importantly, the permeability can be engineered by simple adjustment of the shell hydrogel composition.

It would be beneficial if we could predict the permeability of compartments for a given pore size without performing the customized PCRs. With this goal, we built a physical model considering the free diffusion of semiflexible DNA molecules in porous media (Supporting Information). The model assumes that the diffusible‐to‐confined transition takes place where the geometrical size of DNA is close to the critical dimension of hydrogel pores. In an aqueous solution, the geometry of DNA as a semiflexible polymer can be characterized by its radius of gyration using the Kratky–Porod equation^[^ ^32^ ^]^ (Equation (S1), Supporting Information). Based on this, we derived the relationship between the critical transition length of DNA and *D*_p_ (Equation (S4), Supporting Information). Figure 3c plots model‐defined diffusible and confined regimes divided by Equation S4 (Supporting Information)). For comparison, we included the experimental data derived from Figure 3b. As can be seen, the model exhibits reasonable agreement with the experimental results. Therefore, we believe that the established model can provide a preliminary guide for permeability engineering of the compartments.

### 2.4 Establishing PeCS‐Based CFPS

**Figure 4:**
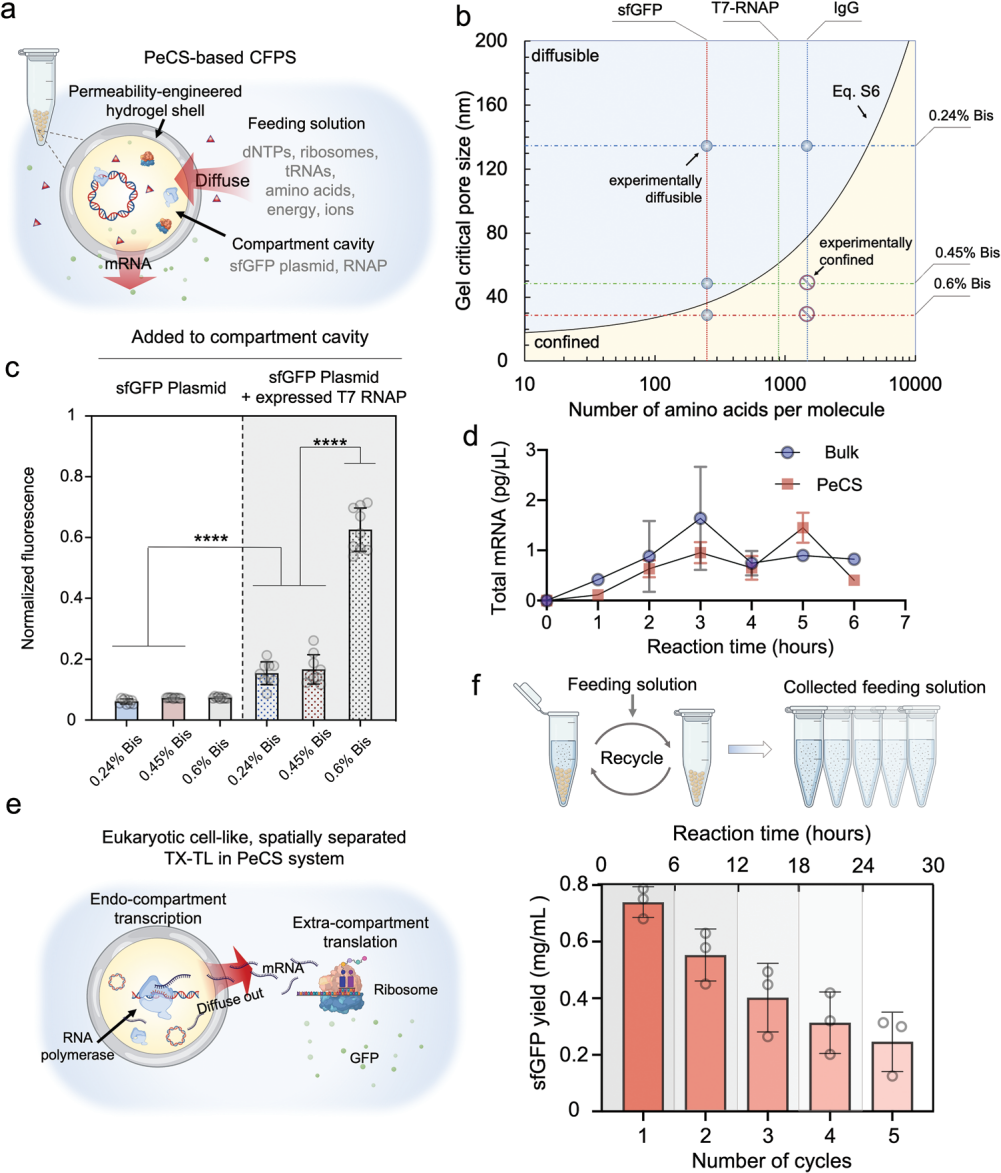
PeCS‐based cell‐free protein synthesis is sustained and recyclable. a) Schematic of the compartmentalized protein synthesis system. Hollow Bis–PAAm compartments encapsulating plasmid and RNA polymerase (RNAP) are immersed in a feeding solution containing other required reagents for RNA transcription and protein translation. b) A physical model describing the transport characteristic of globular proteins across the compartments. The dash‐dotted lines represent the critical pore size of three different Bis–PAAm gels. Vertical dotted lines indicate the total amino acid number of the stated protein molecules. The scattered symbols represent the experimental results verifying the diffusion capability of respective proteins in the compartments (Figure S10, Supporting Information). c) sfGFP fluorescence levels (normalized) of protein synthesis assays performed on three different Bis–PAAm compartments. In the first group (bright background) of experiments, solely sfGFP plasmid was added to the compartment cavity. *E. coli* cell extract containing in vivo expressed T7 RNAP was added along with sfGFP plasmid in the second group (gray background). Error bars denote standard deviations of multiple parallel experiments (*n* ≥ 8). d) Total mRNA levels during sfGFP expression by a PeCS‐based system and a bulk CFPS system. Error bars denote standard deviations of multiple parallel experiments (*n* = 3). e) Proposed transcription–translation procedure in the PeCS‐based system. Transcription and translation are spatially separated. Plasmids and RNAP are confined in the compartments for transcription. The produced mRNAs transport to the feeding solution where translation takes place. f) Workflow (left) and yield (right) of multicycle sfGFP synthesis by a PeCS‐based system. For each cycle, the 0.6% Bis–PAAm compartments containing sfGFP plasmid and cell extract were incubated in a fresh feeding solution for 6 h, after which compartments were magnetically recycled and the sfGFP yield in the supernatant was quantified (*n* = 3).

Having explored the configurability of the compartments, we next sought to reconstitute PeCS‐based cell‐free protein synthesis. Typical CFPS assays are one‐off reactions where synthesized protein products and the molecular machinery are all mixed together.^[^ ^27^ ^]^ The latter is not recyclable and often discarded in subsequent protein purification steps, which limit the total cost‐effectiveness and disobey the central goal of green biosynthesis. In nature, eukaryotic cells envelop the genome and gene transcription activities in its nucleus. The nucleus is permeable to RNA transcripts, allowing them to transport to the cytoplasm and therefore can the protein translation occur.^[^ ^33^ ^]^ We aimed to establish a similar spatially decoupled TX–TL with the PeCS system in which the transcription activities are confined in the compartments. In this configuration, key molecular components such as plasmids and RNA polymerase (RNAP) can be recycled along with the compartments after a complete round of CFPS. As shown in **Figure** **4**a, the system consists of hollow compartments containing gene plasmids and RNAPs, and a feeding solution including other materials for the TX–TL. We hypothesized that by engineering compartment permeability, the plasmids and RNAP could be retained within the core cavity (≈ 110 pL in volume). Other required materials, such as deoxynucleotides (dNTPs) and energy, could continuously diffuse into the cavity to supply the transcription. Driven by the concentration gradient, the transcribed messenger RNA (mRNA) could transport to the feeding solution for the translation to occur. With the established DNA transport model (Figure 3c), we could expect that a 2.48 kb plasmid encoding sfGFP would be confined in the compartments. Even taking supercoiling into account does the physical size of the plasmid exceed the largest pore size in our case.^[^ ^34^ ^]^ To predict protein transport across the compartments, we modified the DNA model by replacing the gyration radius of DNA with that of a globular protein molecule given its total number of amino acids, which led to a brief model describing the diffusible‐to‐confined characteristics of proteins (Figure 4b; Equation (S6), Supporting Information). To verify the model, we experimentally tested the diffusion of sfGFP (26.8 kDa, 238 amino acids) and immunoglobulin G (IgG; ≈150 kDa, ≈1300 amino acids) through the compartment shells (Figure S10, Supporting Information). The results (marked in Figure 4b) are in reasonable accordance with the model. For CFPS, the model suggests that T7 RNAP, comprising 883 amino acids, would be confined in 0.6% Bis–PAAm compartments featuring the smallest pores (≈31 nm) and the 0.45% Bis–PAAm compartment is just around the boundary. Note that *Escherichia coli* ribosome is a vast RNA–protein complex, of which the number of amino acids exceeds 7000.^[^ ^35^ ^]^ Thus, we expected that ribosomes could not transport through all of these compartments.

With insights gained from the models, we moved to experiment with PeCS‐based CFPS. We first encapsulated sfGFP plasmids solely in the compartments and incubated ≈100 000 compartments in a 45 µL *E. coli* extract‐based CFPS mixture. The resultant sfGFP fluorescence is weak for all the gel compositions (Figure 4c), suggesting that the TX–TL procedure could barely occur. This is possibly attributed to the compartment shell's physical barrier effect, which limits the effective transport of RNAP into the compartment cavity. Bio‐macromolecules such as proteins and DNAs exhibit significantly lowered diffusion coefficients when confined in hydrogels due to the physical interactions.^[^ ^36^ ^]^ Although our model suggests that RNAP could migrate into at least the 0.24% Bis–PAAm compartments, the restricted diffusion dynamics might lead to limited local concentration and thus low transcription efficiency in all those compartments. Besides, the migration of RNAPs in the core cavity could also be slowed down as a result of macromolecular crowding considering the plasmids and potentially the de‐crosslinked PAAm chains being trapped in the cavity. These factors suggest that the RNAPs could hardly migrate in the compartments but might be easier to diffuse out. Therefore, in the next experiment, we directly added in vivo expressed T7 RNAP to the core cavity and observed notable increases in sfGFP fluorescence. Notably, the fluorescence level of 0.6% Bis–PAAm compartments is significantly elevated, 8.5‐folds of the previous value where only plasmid was added and over 3.5‐folds of those compartments with larger pores (0.24% and 0.45% Bis). The sfGFP yield was 0.74 mg mL^−1^ for 0.6% Bis–PAAm compartments (also see Figure S13b in the Supporting Information for comparison with the bulk system), comparable to the performance of recently reported open CFPS systems.^[^ ^37^ ^]^ Several findings can be concluded from these experiments: 1) mRNAs indeed diffused to the feeding solution (otherwise, sfGFP cannot be expressed); 2) retaining RNAPs in the limited cavity space could vastly facilitate the transcription; 3) RNAPs were confined in 0.6% Bis–PAAm compartments, thus achieving a significantly increased protein expression level, which is in reasonable agreement with the model. For further confirmation, we monitored the mRNA levels in the feeding solution (Figure 4d). It can be seen that mRNA expression in the PeCS system was slightly delayed compared to a bulk CFPS assay, possibly due to the process of restricted mRNA transport. The two levels became comparable when both systems reached equilibrium (after 4 h). Moreover, we added an extra dose of RNAP to 0.6% Bis–PAAm compartments and observed a further increased sfGFP yield (Figure S13a, Supporting Information). These results signify the successful establishment of a PeCS‐based high‐efficiency CFPS system. Uniquely, the system decouples transcription and translation in physical space (Figure 4e). Through permeability engineering, the compartments retain transcription events in the core cavity. The translation events occur in the feeding solution, triggered by the RNA transcripts migrating through the compartment shell. Such a complex and regulable TX–TL process is similar to the in vivo process of eukaryotic cells, which is previously unavailable to reconstruct in vitro.

### 2.5 Multicycle Protein Synthesis with PeCS‐Based CFPS

Having established the PeCS‐based CFPS system, we wanted to test whether our proposed goal‐sustained and multicycle protein synthesis could be achieved. To do this, we encapsulated sfGFP plasmids and RNAP in 0.6% Bis–PAAm compartments, which were subsequently immersed in the feeding solution for protein synthesis. After each cycle, the feeding solution was collected, and the compartments were magnetically extracted and added to a new feeding solution. We conducted a five‐cycle protein synthesis and quantified the yield of each cycle (Figure 4f). The overall yield of the five‐cycled expression exceeded 2.2 mg mL^−1^, around three folds of its first cycle. The decrease in protein yield along the cycles is possibly attributed to the reduced catalytic efficiency of RNAP after prolonged functioning. To investigate this, we monitored the mRNA levels at the end of each cycle (Figure S13c, Supporting Information). However, given that mRNA degrades quickly (half‐life ≈ 5 min for *E. coli*),^[^ ^38^ ^]^ the levels recorded at the termination of each cycle (after 6 h upon expression) are comparatively low with respect to the levels measured during the reaction in Figure 4d and do not show reasonable correlation with the yield (in Figure 4f). Even so, these results have adequately verified that we achieved multicycle in vitro protein synthesis, a step toward green and sustainable biosynthesis.

### 2.6 Establishing PeCS‐Based Whole‐Cell Biosensors

**Figure 5:**
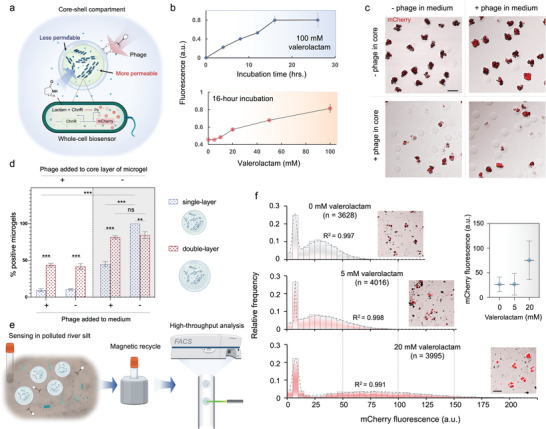
PeCS‐based whole‐cell biosensors resist lytic bacteriophages and exhibit increased fitness in complex environments. a) Schematic of the compartmentalized biosensor. An engineered *E. coli* strain is encapsulated in the cores (1% agarose) of layered compartments for cell growth and functioning. The strain senses an industrial chemical pollutant—lactam—through a constructed lactam–mCherry pathway. A dense outer layer (2% agarose) protects the encapsulated biosensor strain from environmental impacts such as phage attacks. b) Characterization of the compartmentalized biosensors. Top: fluorescence of individual biosensor compartments (*n* ≥ 50) versus incubation time. The compartments were cultured in a medium containing 100 mm valerolactam. Bottom: dose–response relation of the compartmentalized biosensors (*n* ≥ 50). c) Optical microscopic images showing growth condition of biosensor strain encapsulated in core–shell compartments in the presence of phage. The compartments were incubated in a medium containing 5 mm valerolactam for 16 h. The bacteria colonies are labeled in red as they express mCherry fluorescent protein. d) Comparison of biosensor strain growth in single‐layer agarose (1%) beads and in double‐layer compartments in the presence of phage. Microgels having grown colonies were considered positive. Error bars denote standard deviations of parallel experiments (*n* = 3). e) Workflow of sensing, retrieval, and analysis of the compartmentalized biosensors in an artificial river silt environment polluted by lactam. f) Fluorescence profile of compartments after sensing varying levels of valerolactam in polluted river silt. Dashed curves denote dual‐peak Gaussian fitting. The microscopic images display representative compartments at relevant biosensing conditions. The inset scatter plot shows the mean and standard error of corresponding sensing results. Scale bars: 100 µm.

We next attempted to compartmentalize and create a higher‐hierarchy system—bacteria whole‐cell biosensors. Synthetic biology has rendered genetically modified microorganisms (GMMs) capable of living diagnostics and therapeutics,^[^ ^39^ ^]^ programmable living materials,^[^ ^40^ ^]^ and environmental sensing.^[^ ^41^ ^]^ However, the introduction of genetic circuits exerts an extra metabolic burden, which decreases the GMM's fitness in complex environments.^[^ ^42^, ^43^ ^]^ With PeCS, we aimed to provide a deployable solution to this challenge by encapsulating GMMs (a whole‐cell biosensor strain) in layered microgels with a permeability gradient: the core gel matrix is coarse, which provides a cozy environment for cell growth and function; the shell matrix is fine, barring external microbial competitors and predators (**Figure** **5**a). As a demonstration, we explored the core–shell architecture (50 µm core diameter and 10 µm shell thickness) to accommodate lactam biosensing *E. coli* cells. The core is composed of coarse and high‐permeability agarose gel favorable for cell growth and biosensing. The shell is a layer of low‐permeability agarose matrix that bacteria and even smaller particles (e.g., phages) could hardly penetrate. Lactam is an essential class of nylon precursors and can be detected in the environment as an industrial chemical pollutant.^[^ ^44^ ^]^ The concentration of lactam residual in the wastewater falls within 100 mm range.^[^ ^45^ ^]^ To build lactam‐responsive biosensors, we transformed a lactam‐sensing plasmid into *E. coli* DH5*α*. The plasmid carries a ChnR–Pb transcription factor–promoter pair that drives mCherry expression upon recognizing lactam by the ChnR protein.^[^ ^46^ ^]^ The encapsulated cells exhibited excellent colony formation and lactam‐sensing behavior (Figure 5b). These PeCS‐based living sensors display a dynamic window of 5–100 mm valerolactam and a response time of ≈16 h with no noticeable signal degradation up to 26 h, in reasonable relevance with the environmental pollutant levels. For the whole‐cell biosensors to distinguish valerolactams in 0–5 mm, extra efforts in metabolic engineering may be required.^[^ ^41^ ^]^ These results suggest that the PeCS core–shell architecture supports the long‐term growth and functioning of GMMs.

### 2.7 PeCS‐Based Whole‐Cell Biosensors Resist Lytic Phages

We further investigated whether permeability‐engineered PeCS compartments could provide adequate protection against phage attacks. Wild DH5*α* lytic phages were enriched from swine wastewater samples collected at a farm in rural Shanghai. A plaque assay confirmed the phage's lytic activity on the engineered biosensor strain (Figure S15, Supporting Information). We then added the phages to the culture medium and/or the gel core of the compartments and examined the cell growth. As depicted in Figure 5c, colonies formed in most of the compartments when the phages were absent. When the phages were only present in the medium, the biosensor colonies formed as normal. By contrast, the frequency of colonies dropped significantly when the phages were present in the core of compartments. The results suggest that 1) the phages can lyse the bacteria when present in the gel core, and 2) phages in the medium cannot enter the compartments. To further confirm that it was the shell layer that barred the phages, we compared the biosensor growth in bare core beads versus in the core–shell system (Figure 5d; Figure S16, Supporting Information). As seen, both systems functioned comparably in the absence of phage. With phages in the medium, however, colonies were only found in less than half of the single‐layer beads, whereas the impact was negligible for the core–shell compartments. Even when phages were present in the core gel, over 40% of the compartments were found positive. By contrast, the positive rate of single‐layer beads was less than 10%. The results reveal that 1) it is the shell layer that resists the phages, not the more permeable core; 2) the core–shell architecture may offer a bidirectional protective effect that not only resists the phages from outside but also prevents the leakage of phages from inside, thus limiting the spread rate of phages between the compartments. Overall, the experiments verify that permeability‐engineered core–shell PeCS compartments can protect the encapsulated bacteria biosensors against phage invasion.

### 2.8 Sustained Function and Retrieval of PeCS Whole‐Cell Biosensors in Complex Environments

To further explore the reconstituted biosensors to survive in ill‐defined surroundings, record and memorize desired information, and be retrieved for reading the stored information, we designed an experiment to sense lactam in a complex environment (Figure 5e). The biosensor compartments were put in artificial river silt samples polluted by certain levels of valerolactam. After 32 h, they were magnetically purified from the river silts and then subjected to flow cytometry to extract the stored information. Preliminary culture experiments reveal the competition between native microbes and the introduced GMMs (Figure S17, Supporting Information). The biosensor growth was found markedly slowed down without applying strain‐specific antibiotics, yet a duration of 32 h was found to be sufficient for the biosensors to grow and function. The flow cytometry results in Figure 5f display the biosensor response (fluorescence) to various pollutant levels extracted from thousands of retrieved PeCS compartments. Gaussian fitting was performed on the data, showing that each distribution diagram features two distinct peaks. The first peaks fixed at lower fluorescence (5–10 a.u.) were considered to represent empty compartments or debris. Therefore, we derived the fluorescence profile from the second peaks as the effective output (Figure 5f, inset). Clearly, the compartmentalized biosensors successfully recorded corresponding valerolactam levels even in such a complex environment. Notably, the recorded results are consistent with biosensor performance in a culture medium (Figure 5b). This suggests that re‐calibration may not be required when the biosensors are adopted to a strange environment. The above results prove that permeability‐engineered compartmentalization enables a complete round of living‐cell‐based biosensing in a close‐to‐reality setting: survival, growth, recording, and retrieval.

## 3 Discussion

### 3.1 Advantages and Potential Applications of PeCS

First of all, PeCS offers a new dimension of configurability—compartment permeability. The regulation of DNA, protein, and bacteriophage transport has been demonstrated in the above experiments. Based on simple size selection, the regulation should be expandable to various biomolecules and particles below 100 nm, such as biological/artificial enzymes, small unilamellar vesicles, and viruses, to enable more complicated applications. Next, PeCS compartments are configurable in materials and architecture, which has led to the strategy's broad compatibility and several other benefits: 1) the compartments can be magnetized by magnetic NPs’ (MNPs) encapsulation, facilitating sample purification and retrieval; 2) likewise, the hydrogel‐based core and shell can be further decorated (e.g., with antibodies and nucleic acid probes) to enable new functionalities; 3) with proper tuning of the outer diameter, the compartments are compatible with commercial instruments such as a flow cytometer (typically accept particle size below 60 µm) and a digital PCR reader (require particle size ≈ 90 µm), making the profiling of millions of compartments possible.

Through permeability engineering, PeCS has enabled various biochemical assays such as regulable nucleic acid amplification and decoupled TX–TL, and cell activities including growth and long‐lasting functioning. The combination of these existing assays and their further extension will lead to novel applications that are previously challenging. For example, PeCS should allow a large library of gene mutants to be amplified and the encoding proteins to be synthesized in millions of individual compartments; then, the compartments can be sorted (e.g., based on fluorescent signals related to enzymatic activity) to extract the gene sequences, thus enabling ultrahigh‐throughput cell‐free protein evolution. Moreover, similar workflows can be used to evolve and select microbe strains for high‐yield biosynthesis.

The demonstrated phage resistance and robustness of PeCS should render many synthetic biology applications practical.^[^ ^47^ ^]^ For instance, unculturable microbes can be encapsulated in PeCS compartments, delivered to the original environment (e.g., gut and soil), cultured and re‐collected (by magnetic purification), potentially enabling efficient amplification of hard‐to‐culture microbes for microbiome study. A similar workflow may allow encapsulated GMMs to detect and record environmental signals in the mammalian gut and be retrieved to extract the stored information.^[^ ^43^ ^]^ Moreover, the workflow should also allow drug‐releasing bacteria to grow in the gut, release the therapeutic biomolecules while still being contained, and be retrieved to prevent interfering with the original microbiome, thus enabling bacterial cancer therapies.^[^ ^48^ ^]^ For synthetic biology in agriculture, PeCS should render GMMs’ field applications friendlier, enabling potential applications such as soil environmental sensing,^[^ ^49^ ^]^ soil fertility control,^[^ ^50^ ^]^ and pest management.^[^ ^51^ ^]^ Beyond synthetic biology, PeCS should be useful in parallel biochemical assays. For instance, PeCS could enable millions of cells to be encapsulated and lysed, and their genomes to be amplified, probed, and perhaps sequenced.

### 3.2 Limitations and Future Improvements

While our strategy possesses notable advantages, the current manufacturing scheme might impose several limitations. First, the compartment could occasionally break, releasing biological materials in the cavity to the outer environment, ending with the competition for reaction substrates. This could be addressed by introducing dual‐crosslinked tough hydrogels as the shell material. Second, unlike liposome or cellular membrane, where the core and the outer environment could maintain different liquid compositions, the porous nature of hydrogel precludes the establishment of chemical gradient, as is the case in some biological active transport processes. Besides, the tuning of selective permeability solely relies on adjusting hydrogel composition during the encapsulation step. It would be favorable if the permeability could be controlled more actively after the compartment formation. A current investigation is underway to incorporate temperature‐ or photo‐responsive hydrogels^[^ ^52^ ^]^ in the PeCS system.

## 4 Experimental Section

Materials and methods for this study are included in the Supporting Information.

## Conflict of Interest

The authors have filed a provisional patent based on this work.

## Author Contributions

L.L. and R.Z. contributed equally to this work. Y.L., J.Z., and J.L. directed the research; Y.L., J.Z., and J.D.K. conceptualized the study. Y.L., J.Z., L.L., and R.Z. designed the experiments. L.L., R.Z., and L.C. carried out the experiments. X.T., X.J., and F.B. assisted in the cell‐free protein synthesis experiments. B.P., C.M., T.L., C.X., and Y.S. contributed to the biosensing experiments. Y.L., L.L., R.Z., J.L., J.Z., X.M., and J.D.K. analyzed the data and discussed the results. Y.L., J.Z., and L.L. wrote the manuscript with help from all authors.

## Supporting information

Supporting Information

## Data Availability

The data that support the findings of this study are available from the corresponding author upon reasonable request.
